# The Impacts of a Mentoring Program on the Development of Gerontological Social Work Faculty

**DOI:** 10.1093/geroni/igaa057.1796

**Published:** 2020-12-16

**Authors:** Nancy Kusmaul, Stephanie Wladkowski, Allison Gibson, Rebecca Mauldin, Jennifer Greenfield, Noelle Fields

**Affiliations:** 1 UMBC, Baltimore, Maryland, United States; 2 Eastern MIchigan University, Ypsilanti, Michigan, United States; 3 University of Kentucky, College of Social Work, Lexington, Kentucky, United States; 4 University of Texas at Arlington, Arlington, Texas, United States; 5 University of Denver, Denver, Colorado, United States; 6 The University of Texas at Arlington, Arlington, Texas, United States

## Abstract

The John A. Hartford Foundation and the Association for Gerontology Education in Social Work (AGESW) have worked to develop gerontological social work faculty to address the needs of older adults. This presentation will discuss the role of AGESW’s Pre-Dissertation Fellows Program in the development of social work doctoral students. All participants from the PDFP’s 2010-2016 cohorts received a 38-question online survey via email exploring the program’s impacts on their academic career in teaching, research, mentoring, and support. Forty-five respondents, representing all six cohorts, completed the survey. More than half said the PDFP contributed to their ability to publish research (64.4%, n = 29), grow their professional network (86.7%, n = 39), and teach (55.5%, n = 25). Doctoral programs provided different experiences: mentoring, methodological training, professional development, and peer support. Results suggest the PDFP supplements students’ doctoral programs by connecting students to each other and to national leaders.

